# Development and Validation of a *Bordetella pertussis* Whole-Genome Screening Strategy

**DOI:** 10.1155/2020/8202067

**Published:** 2020-04-02

**Authors:** Ricardo da Silva Antunes, Lorenzo G. Quiambao, Aaron Sutherland, Ferran Soldevila, Sandeep Kumar Dhanda, Sandra K. Armstrong, Timothy J. Brickman, Tod Merkel, Bjoern Peters, Alessandro Sette

**Affiliations:** ^1^Division of Vaccine Discovery, La Jolla Institute for Immunology, La Jolla, San Diego, California, USA; ^2^Department of Microbiology and Immunology, University of Minnesota Medical School, Minneapolis, Minnesota, USA; ^3^Division of Bacterial, Parasitic and Allergenic Products, Center for Biologics Evaluation and Research, U.S. Food and Drug Administration, Silver Spring, Maryland, USA; ^4^University of California San Diego School of Medicine, La Jolla, San Diego, California, USA

## Abstract

The immune response elicited by the protective whole-cell pertussis (wP) versus the less-protective acellular pertussis (aP) vaccine has been well characterized; however, important clinical problems remain unsolved, as the inability of the currently administered aP vaccine is resulting in the reemergence of clinical disease (i.e., whooping cough). Strong evidence has shown that original, childhood aP and wP priming vaccines provide a long-lasting imprint on the CD4+ T cells that impacts protective immunity. However, aP vaccination might prevent disease but not infection, which might also affect the breadth of responses to *Bordetella pertussis* (BP) antigens. Thus, characterizing and defining novel targets associated with T cell reactivity are of considerable interest. Here, we compare the T cell reactivity of original aP and wP priming for different antigens contained or not contained in the aP vaccine and define the basis of a full-scale genomic map of memory T cell reactivity to BP antigens in humans. Our data show that the original priming after birth with aP vaccines has higher T cell reactivity than originally expected against a variety of BP antigens and that the genome-wide mapping of BP using an ex vivo screening methodology is feasible, unbiased, and reproducible. This could provide invaluable knowledge towards the direction of a new and improved pertussis vaccine design.

## 1. Introduction

Several studies and epidemiological evidence suggest that the immunity induced by *Bordetella pertussis* (BP) acellular vaccines (aP) wanes more rapidly as compared to the immunity elicited by vaccines based on whole BP cells (wP) [1–8]. In previous studies [9, 10], we have investigated potential immune correlates of this waning immunity, by dissecting immune responses in young adults, originally primed with either aP or wP vaccines. These studies were enabled by the definition of the T cell epitopes contained in the BP antigens contained in the aP vaccines (pertussis toxin, PtTox; two serotypes of fimbriae, Fim2/3; filamentous hemagglutinin, FHA; and pertactin, PRN), which was achieved following the systematic analysis of responses following the expansion of antigen-specific T cells in short-term in vitro culture [9], and the development of the activation-induced marker (AIM) assays, which allowed measurement of responses directly ex vivo without any further manipulation [11, 12].

When immune responses to aP boosters in individuals who received their initial doses with either wP or aP vaccines were compared, BP-specific memory CD4^+^ T cell responses were associated with Th1/Th17 versus Th2 differential polarization as a function of childhood vaccination [10]. Strikingly, after aP booster, donors originally primed with aP were associated with lower responses ex vivo and lower in vitro proliferation. These observations led to the hypothesis that lower proliferative capacity of aP might be linked to a regulatory cell population, since no difference between cohorts was noted when purified T cell subpopulations were assayed.

As mentioned above, the available data demonstrates that the BP wP vaccine is effective and prevents infection, but the total breath of responses is not known. Based on these observations, it could be expected that broad responses are elicited upon vaccination, directed against a variety of different BP antigens. These responses could be possibly blunted over time with repeated aP boosters. The data present in the literature also demonstrate that the aP vaccine despite waning over time is also effective in initial protection against whooping cough [3, 13–15], but by comparison to the wP vaccine, it could be expected that this vaccine would elicit a narrow response directed mostly against the four vaccine antigens. As a result, little or no response against non-aP vaccine BP antigens would be detected. Several lines of evidence argue against this simplistic view, which are related to the possible interplay between vaccination and natural BP exposure/infection [16]. Specifically, recent data from both baboon and mouse models [15, 17–21] suggests that aP vaccination might prevent disease but not infection or in particular nasopharyngeal subclinical colonization. This might paradoxically result in broad responses in aP and is of greater magnitude than wP because of heavier and more frequent exposure.

To specifically address this, we set out to study T cell responses from BP antigens not contained in the aP vaccine, as function of either aP or wP priming vaccination, as well as to address the feasibility of using a large-scale ex vivo methodology for T cell epitope identification specific for BP.

## 2. Materials and Methods

### 2.1. Study Subjects

We recruited 31 healthy adults from San Diego, USA (Supplementary [Supplementary-material supplementary-material-1]). All participants provided a written informed consent for participation, and clinical medical history was collected and evaluated by the clinical coordinators through questionnaires, recording dates and numbers of vaccination, including the information that no boost was administered in at least the previous four years prior to this study. All donors were from the San Diego area and originally vaccinated with either DTwP or DTaP priming vaccines in infancy (three doses at 2, 4, and 6 months and then two doses between 15-18 months and 4-6 years) and followed the recommended vaccination regimen (which is also necessary for enrollment in the California school system), which entails immunization with the acellular booster vaccine Tdap at 11-12 years and then every 10 years and during pregnancy. The pertussis (P) compounds in these vaccines (“w” for whole-cell, also wP for short, and “a” for acellular, also aP for short) are coadministered with diphtheria toxoid (D) and tetanus toxoid (T). Also, the capital and lowercase letters denote higher or lower proportions of the overall components between vaccines. Individuals who had been diagnosed with BP infection at any given time in their life were excluded. In all groups, male and female subjects were included equally.

### 2.2. Study Approval

This study was performed with approvals from the Institutional Review Board at La Jolla Institute for Immunology (protocols: VD-101-0513 and VD-059-0813). All participants provided a written informed consent for participation, and clinical medical history was collected and evaluated.

### 2.3. Peptides

Peptide selection was derived either from *Bordetella pertussis* (BP) whole-genome predictions from the Tohama I strain or from experimentally validated antigens included in the aP vaccines (FHA, Fim2/3, PRN, and PtTox). Experimentally validated peptides were selected from a total of 785 peptides tested from the BP strain Tohama I, encompassing 16-mers overlapping by eight residues of the full-length coverage of all antigens. The top epitopes recognized by >5% donors corresponding to 132 peptides were chosen, and the megapool (MG) of all combined peptides is described as PT (MG) hereafter [9, 10]. BP genome-wide identification was performed by scanning for the presence of predicted HLA class II promiscuous binding peptides. MHC-peptide binding predictions were performed using publicly available tools hosted by the Immune Epitope Database (IEDB) Analysis Resource [22]. Specifically, the prediction of peptides was established by the 7-allele HLA class II restricted method and by using peptides 15 residues in length and overlapping by 10 residues [23, 24]. Additional filtering using an epitope cluster analysis tool [25] was performed. Briefly, the peptides from each ORF were created in an overlapping fashion, which due to a shared 9-amino acid core prediction base often had similar binding affinities (percentile score). A modified algorithm from IEDB clustering tool to remove redundant peptides sharing a stretch of 9-amino acids in 15-mers was performed. In case of peptides sharing a core, the peptides with highest binding affinities were picked. At least 2 peptides per open reading frame (ORF) were selected. The selected peptides were pooled together and underwent sequential lyophilization as described elsewhere [10] and arranged in “megapools” (MGs) of approximately 200 peptides each further divided into groups of 8 “mesopools” (MSs) of 24 individual peptides. For BP adenylate cyclase toxin (ACT), the full length of the antigen was covered and the top 20% predicted epitopes were selected to originate a 54 peptide ACT MGs. All individual peptides (Supplementary [Supplementary-material supplementary-material-1]) were synthesized by Mimotopes (Victoria, Australia) and resuspended to a final concentration of 1 mg/mL in DMSO.

### 2.4. PBMC Isolation

Peripheral blood mononuclear cells (PBMCs) were isolated from whole blood or leukapheresis by density gradient centrifugation according to the manufacturer's instructions (Ficoll-Paque Plus, Amersham Biosciences, Uppsala, Sweden) as previously described [26]. The cells were cryopreserved in liquid nitrogen and suspended in fetal bovine serum (FBS) containing 10% (vol/vol) dimethyl sulfoxide (DMSO).

### 2.5. *Bordetella pertussis* Lysate Production

BP lysates were prepared from iron-starved bacteria (to simulate growth conditions in the natural mammalian host environment) [27–29], provided by Drs. Sandra Armstrong and Timothy Brickman (University of Minnesota Medical School, USA). Specifically, the bacteria were cultured in the iron-deficient chemically defined Stainer-Scholte medium [30, 31]. The bacterial cells were harvested at mid- to late-exponential growth phase and suspended in phosphate-buffered saline. The lysate was generated by mechanical shearing at low temperature (French press), frozen in liquid nitrogen, and then shipped to the La Jolla Institute for Immunology (CA, USA). Growth was monitored by optical density readings, and the iron starvation status of the bacteria was confirmed using siderophore detection assays [32, 33] and SDS-PAGE analysis of proteins.

### 2.6. AIM Assay

The activation-induced marker (AIM) assay was previously described [12]. This assay detects cells that are activated as a result of antigen-specific stimulation by staining antigen-experienced CD4+ T cells for TCR-dependent upregulation of OX40 and CD25 (AIM25) and/or PD-L1 (AIMPD) after an optimal time of 18–24 h of culture. Briefly, cryopreserved PBMCs were thawed, and 1 × 10^6^ cells/condition were immediately cultured together with PT and ACT peptide pools (2 *μ*g/mL), selected MS (1 *μ*g/mL), individual peptides (10 *μ*g/mL), or PHA (10 *μ*g/mL; Roche) and DMSO as positive and negative controls, respectively, in 5% human serum (Gemini Bio-Products) for 24 h. To determine the memory phenotype of responding T cells, staining for CD45RA and CCR7 markers was performed and subpopulations were defined as follows: naive T cells (Tn): CD45RA+CCR7+; effector memory RA T cells (Temra): CD45RA+CCR7-; T central memory (Tcm): CD45RA-CCR7+; and T effector memory (Tem): CD45RA-CCR7-. The samples were acquired using a BD LSR II flow cytometer (BD Biosciences) and analyzed using the FlowJo X software. Specific signals were all subtracted to the DMSO control displayed as % of CD4 T cells and normalized and displayed as the number of cells per 1 × 10^6^ CD4 T cells. All flow cytometry mAb reagents for surface staining are listed in Supplementary [Supplementary-material supplementary-material-1].

### 2.7. ELISpot and FluoroSpot Assays

Culturing of PBMCs for in vitro expansion was performed by incubating in RPMI (Omega Scientific) supplemented with 5% human AB serum, GlutaMAX (Gibco), and penicillin/streptomycin (Omega Scientific) at 2 × 10^6^ per mL in the presence of BP lysates at 10 *μ*g/mL. Every 3 days, 10 U/mL IL-2 in RPMI medium was added to the cultures. After 14 days in culture with the BP lysate, the expanded T cells were tested for the recognition of the epitope pools or individual peptides as described above. As readout, the standard IFN*γ*/IL-5 and IL-17/IL-9 cytokine combination for ELISpot assay or IFN*γ*/IL-5/IL-13 cytokine combination for FluoroSpot assay was performed as described [9, 34]. For ex vivo determinations, the same combination of cytokines was used after 20 h incubation with lysate (10 *μ*g/mL), epitope pools (2 *μ*g/mL), or individual peptides (10 *μ*g/mL) besides PHA (2 *μ*g/mL) and DMSO as positive and negative controls, respectively. Consistent with these previous studies in order to be considered positive, a response in both in vitro or ex vivo modalities had to match all three different criteria: (1) eliciting at least 20 spot-forming cells (SFC) per 10^6^ PBMCs; (2) *p* ≤ 0.05 by Student's *t*-test or by the Poisson distribution test; and (3) stimulation index (SI) ≥ 2.

### 2.8. Statistical Analysis

Comparisons between groups were made using the nonparametric two-tailed, unpaired Mann–Whitney *U* or Spearman's rank correlation coefficient tests. Prism 8.0.1 (GraphPad) was used for all these calculations. All figure data in which error bars are shown are presented as median ± interquartile range when each dot represents an individual donor or as mean ± SEM when each dot represents a technical replicate. A *p* value < 0.05 was considered statistically significant.

## 3. Results and Discussion

### 3.1. Original aP and wP Primes Are Associated with High T Cell Reactivity for All Antigens Contained and Not Contained in aP Vaccine

There are emerging proofs of BP subclinical colonization in vaccinated individuals, including indirect evidences of asymptomatic transmission [35–38], as observed in animal models [19, 21], which could affect T cell responses against BP antigens to a greater extent than originally expected.

**Figure 1:**
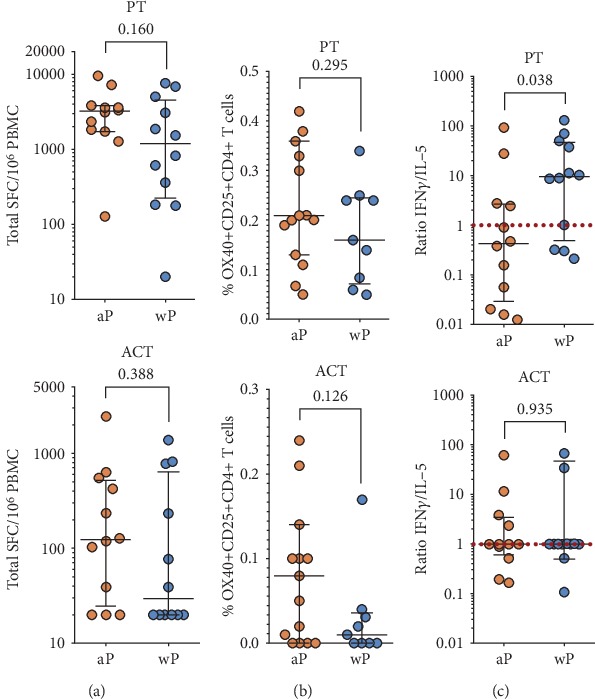
Original childhood aP priming is associated with high reactivity to pertussis antigens. (a) Cytokine production was measured by ELISpot following in vitro restimulation with BP lysate. Magnitude of responses expressed by spot-forming cells (SFC) is shown for the sum of T cell responses (IFN*γ*, IL-5, IL-17, and IL-9) for all aP antigens (PT) or ACT between wP- and aP-primed donors (*n* = 12 for aP and *n* = 12 for wP cohorts). (b) % of BP-specific CD4+ memory T cells for PT or ACT antigens by AIM assay for donors originally primed with wP or aP vaccine (*n* = 15 for aP and *n* = 9 for wP cohorts). (c) Differential polarization of T cell responses for PT but not ACT. Each data point represents the ratio of IFN*γ*/IL-5 SFCs from each donor (*n* = 12 for aP and *n* = 12 for wP cohorts). Data are expressed as median ± interquartile range for each cohort with the Mann–Whitney *U* test comparison value.

To address this issue, we examined memory CD4 responses to a previously defined peptide pool of BP epitopes encompassing the 4 antigens (FHA, Fim2/3, PRN, and PtTox) contained in the aP vaccine [10] in a set of PBMCs derived from 31 donors either originally vaccinated with aP (*n* = 16) or originally vaccinated with wP (*n* = 15). We used two assay formats, one is an ex vivo AIM assay that measures the antigen-specific upregulation of OX40 and CD25 markers and the other is a lysate expansion followed by an ELISpot assay. Using the lysate expansion, we found that responses in the two groups were equivalent (with a small trend towards higher response in the aP group) (Figure 1(a), top). In the same experiments, we also measured responses against a pool of BP epitopes from the adenylate cyclase toxin (ACT) antigen, a virulence factor that is not contained in the aP vaccine [39]. Interestingly, we found that aP donors also exhibited equivalent memory T cell reactivity against the ACT antigen (Figure 1(a), bottom). Importantly, the same results were corroborated when assaying responses in a different subset of donors and using the ex vivo AIM modality (Figure 1(b)). Finally, responses against the set of antigens present in the aP vaccine showed differential polarization as a function of the original priming vaccination consistent with literature [9, 10, 40, 41], but surprisingly, responses against ACT did not (Figure 1(c)). Overall, aP-vaccinated subjects are associated with immune responses against a variety of BP antigens, including antigens not present in the aP vaccine, consistent with the notion that aP prevents disease but not colonization/exposure, and this result is apparent using two different assay modalities.

### 3.2. A Genome-Wide Reactivity Screen of T Cell Reactivity to BP with Different Assay Modalities Considered

The data discussed in the previous section suggests that the definition of a full-scale genomic map of memory T cell reactivity to BP antigens in humans should be both feasible and of considerable interest [42]. Indeed, a similar genome-wide mapping of T cell responses to *Mycobacterial tuberculosis* antigens has been recently accomplished [43].

To explore the feasibility of a similar screen in the case of BP antigens, bioinformatic epitope predictions [42, 44] for the whole BP genome indicated the synthesis of approximately 25,000 peptides, spanning over 3300 BP putative ORFs. Here, we wanted to specifically test the practicality of a potential large-scale screening strategy, by testing a selected set of these 25,000 peptides, which would be initially arranged in approximately 130 “megapools” (MGs) of approximately 200 peptides each. Additionally, we envisioned that each MG would be further divided into 8 “mesopools” (MSs) of about 24 individual peptides. These various pools and peptides would be tested using PBMCs derived from apheresis from 20 young adults originally vaccinated with either wP or aP vaccines.

**Figure 2:**
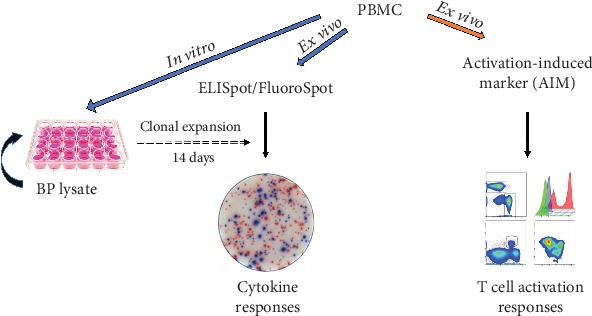
Approaches for genome-wide screening and measurement of T cell reactivity. Schematic design for the screening of BP whole genome using different assay modalities (AIM versus ELISpot/FluoroSpot) and strategies (in vitro versus ex vivo) to capture T cell-specific responses.

Below, we report the results of a pilot study using the selected wP and aP donors to define the screening methodology. We tested 8 MSs containing peptides that have been mapped in the whole BP genome to include the 4 antigens contained in the aP vaccine (PRN, PtTox, Fim2/3, and FHA), but also novel ORFs, not yet assayed for T cell reactivity (Supplementary [Supplementary-material supplementary-material-1]). As a positive control, we tested the previously described PT MG [10], produced with the previously identified vaccine-reactive epitopes and containing 132 peptides. The purpose of the experiments was to specifically compare the results obtained with different screening modalities, including the ex vivo ELISpot*/*FluoroSpot [43], the 14-day restimulation ELISpot*/*FluoroSpot [9], and the ex vivo AIM assay [12] (Figure 2).

### 3.3. In Vitro Lysate Stimulation Allows Identification of BP-Specific Responses

In the first series of experiments, we evaluated the FluoroSpot assay platform ex vivo. When we tested IFN*γ*, IL-5, IL-13, IL-17, IL-9, and TNF-*α* production in response to the 8 MSs, no positive responses were detected. The PT MG composed of known epitopes and used as a positive control also did not elicit any detectable responses (not shown). These results show that, in the case of BP, direct ex vivo screening is not feasible with the FluoroSpot platform as reported for mycobacterial tuberculosis [43].

**Figure 3:**
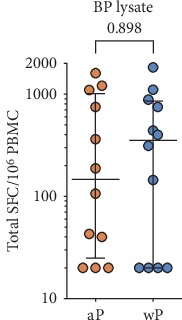
BP lysates can be used to expand cells for assessing BP antigen reactivity. The magnitude of responses expressed by spot-forming cells (SFC) is shown for the sum of T cell responses (IFN*γ*, IL-5, IL-17, and IL-9) for lysate restimulation after 14 days of expansion with BP lysate (*n* = 12 for aP and *n* = 12 for wP cohorts). Data are expressed as median ± interquartile range for each cohort with the Mann–Whitney *U* test comparison value.

As an alternative, and similar to the assay shown in Figure 1(a), we considered an in vitro restimulation step, to expand antigen-specific T cells. In fact, this assay modality has been successfully utilized in numerous studies where allergen extracts were employed to expand allergen-specific T cells [45–48]. In this context, we wanted to determine whether a BP lysate could be also utilized to expand BP-specific T cells and measure BP-specific responses. Accordingly, we produced a lysate prepared from iron-starved BP cultures. PBMCs were stimulated in vitro for 14 days with 10 *μ*g/mL of the lysate, and the expanded T cells were tested in a 24 h FluoroSpot assay, where the number of cells secreting the various cytokines was recorded. The results of a pilot study utilizing PBMCs from 12 aP and 12 wP donors, respectively, are shown in Figure 3, demonstrate that the BP lysate can be used to expand cells for subsequent use in the 14-day restimulation FluoroSpot assay.

### 3.4. In Vitro Lysate Stimulation Allows Identification of Novel BP Antigens and Epitopes

**Figure 4:**
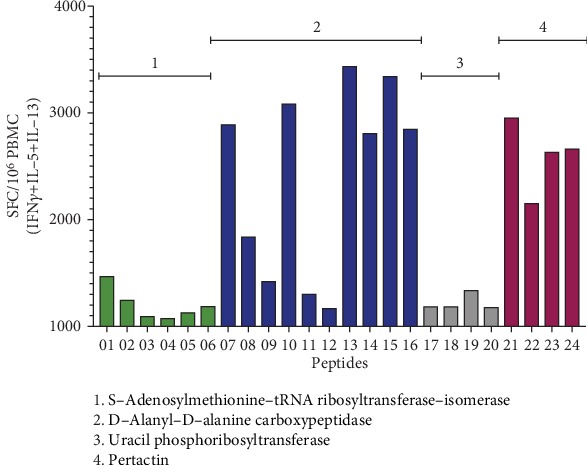
Deconvolution of positive MS reidentifies known antigens and identifies epitopes from novel antigens. Cytokine production was measured by FluoroSpot after 14 days of in vitro restimulation with BP lysate. The magnitude of responses expressed by spot-forming cells (SFC) is shown for the sum of T cell responses (IFN*γ*, IL-5, and IL-13) of a representative donor for all individual peptides (*n* = 24) from a positive mesopool containing the known pertactin antigen. The name of the all the antigens encompassing the group of peptides is shown.

In the next series of experiments, with the method established above, we assayed in vitro cultures from two independent donors, and after the 14-day lysate expansion, the 8 MSs were tested in the FluoroSpot assay for the most abundant cytokine production (i.e., IFN*γ*, IL-5, and IL-13). The MS encompassing peptides from known and novel antigens were all associated with positive signals in the restimulation FluoroSpot assay as well as the positive control (not shown). Representative data are shown for one of the two donors and one of the MSs, where the signal was deconvoluted to map the individual peptides recognized by the responding T cells (Figure 4).

The results indicate that deconvolution of positive MS reidentified the known antigens; in the case of the representative data shown, a vigorous pertactin response was observed. Most importantly, the results indicate that the approach also identifies epitopes from *novel* antigens endowed with high immunoreactivity. Specifically, strong responses were noted for peptide epitopes derived from DD-transpeptidase antigen, a bacterial enzyme involved in cell wall biosynthesis [49]. Further analysis indicated that the MS responses observed were associated with the expected polarization pattern (Th2 for aP, Th1/Th17 for wP) when performing parallel intracellular staining assays (not shown). In conclusion, these results indicate that the in vitro restimulation combined with a FluoroSpot assay is a suitable methodology for the large-scale BP screen.

### 3.5. Ex Vivo *AIM* Assay Reproducibly Identifies Candidates for Deconvolution

**Figure 5:**
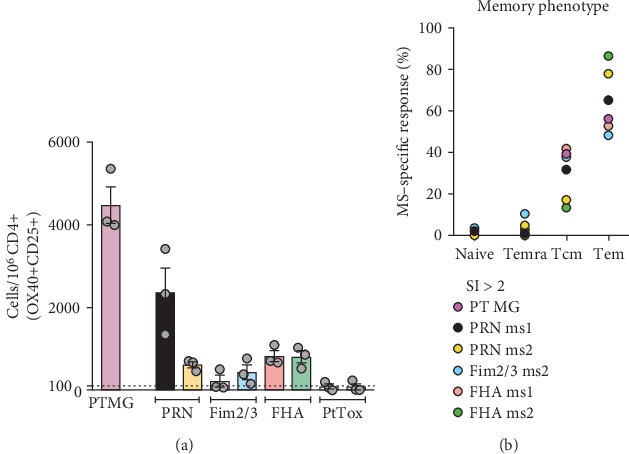
Ex vivo AIM assay pool screening on different days reproducibly identifies candidates for deconvolution. (a) % of BP-specific CD4+ T cells for the pool of aP antigens (PT) or for the 8 MSs containing peptides from the 4 individual aP antigens was measured by the AIM assay. Peptides from the 4 individual antigens are aligned by ORF position but flanked by peptides from other antigens and unequally distributed over 2 adjacent and unique MSs each. Individual circles represent an independent technical replicate of the same donor performed on a different day. A representative donor is shown. Data are expressed as mean ± SEM. Dotted line indicates positive response threshold of 100 cells set to identify MS candidates for deconvolution. (b) Memory subset composition of PT MG- and MS-specific responses (SI > 2) is shown as % of total CD4+ T cells gated in AIM-positive cells (Tn: CD45RA+CCR7+, Temra: CD45RA+CCR7-, Tcm: CD45RA-CCR7+, and Tem: CD45RA-CCR7-). The average data for each positive mesopool are shown (*n* = 5).

In the next series of experiments, we evaluated the feasibility of a screening modality utilizing an ex vivo AIM assay [10–12] similar to the approach used in Figure 1(b). The results of AIM assays where PBMC from a representative donor out of 6 donors were stimulated with the eight MSs are shown in Figure 5(a). The results show data obtained in three completely independent experiments on different days. The results between different days were highly correlated with high significance (*p* < 0.0001) when performing a multiple linear regression analysis, regardless of whether the absolute positive cells/10^6^ cells or SI (signal to noise ratios) were considered. Based on these results, a threshold of 100 cells and SI of 2 were provisionally set to identify MS candidates for deconvolution.

Parallel experiments investigated the memory phenotype of the responding cells, defined on the basis of the expression of the CD45RA and CCR7 markers as described in more detail in Materials and Methods. As expected and in a similar fashion to PT MG composed of known epitopes, the BP-specific T cells detected in the reactive MS (SI > 2) were mostly derived from the effector memory (Tem) and central memory (Tcm) subsets (Figure 5(b)).

**Figure 6:**
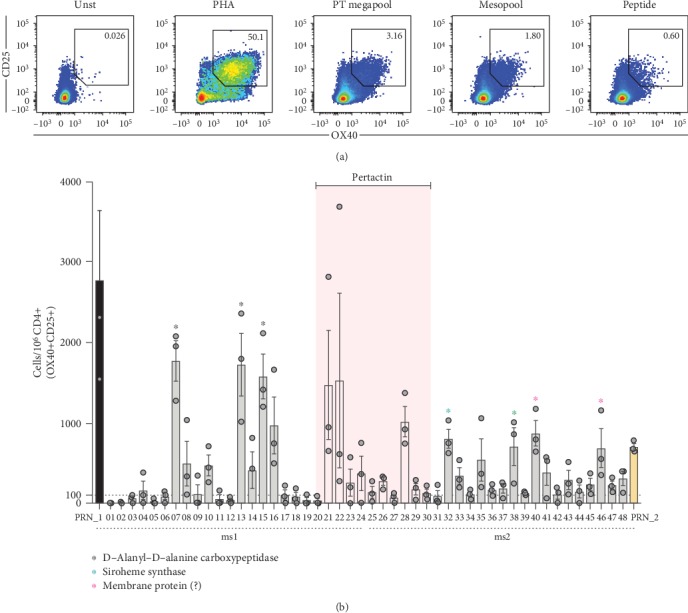
Ex vivo AIM assay can reproducibly detect signals down to the peptide level. (a) Representative flow cytometry plots of CD25+OX40+ upregulation by CD4+ T cells in cells left unstimulated (Unst) or stimulated with pools of peptides (PT-megapool (*n* = 132 peptides) or mesopool (*n* = 24 peptides)) or individual peptides as well as PHA as a positive control. (b) % of BP-specific CD4+ memory T cells for each individual peptide (*n* = 48) deconvoluted from 2 contiguous positive MSs (PRN_1 (ms1, *n* = 24) and PRN_2 (ms2, *n* = 24)) containing among others, peptides (*n* = 10) from the known antigen pertactin (PRN; pink area) as measured by AIM assay. Each dot represents an independent technical replicate of the same donor performed on a different day. A representative donor is shown. Data are expressed as mean ± SEM. The names of the all the antigens encompassing the group of peptides whose responses were significant are shown.

Finally, we investigated whether the two positive MSs containing the pertactin antigen could be further deconvoluted, down to the level of individual peptides. The results shown in Figure 6 show that this is indeed the case. The deconvolution identified peptides from the known aP antigen pertactin and in addition identified epitopes from novel antigens. Specifically, strong responses were noted for peptide epitopes derived from the D-alanyl-D-alanine transpeptidase and siroheme synthase antigens, both enzymes associated with BP biosynthetic processes. The graph shows the results from three independent assays performed on different days and indicates that the assay results are associated with minimal day-to-day variability and high correlation (*p* < 0.0001). Further experiments also revealed a good correlation between the results of the ex vivo AIM assay and cytokine production by the Ag-reactive T cell enrichment (ARTE) assay ([50]; not shown) suggesting that peptide stimulation captures activated cells that are actively producing cytokines. In conclusion, these results indicate that the ex vivo AIM assay is also a suitable methodology for the large-scale BP screen.

### 3.6. Ex Vivo and In Vitro Assays Are Highly Correlated

**Figure 7:**
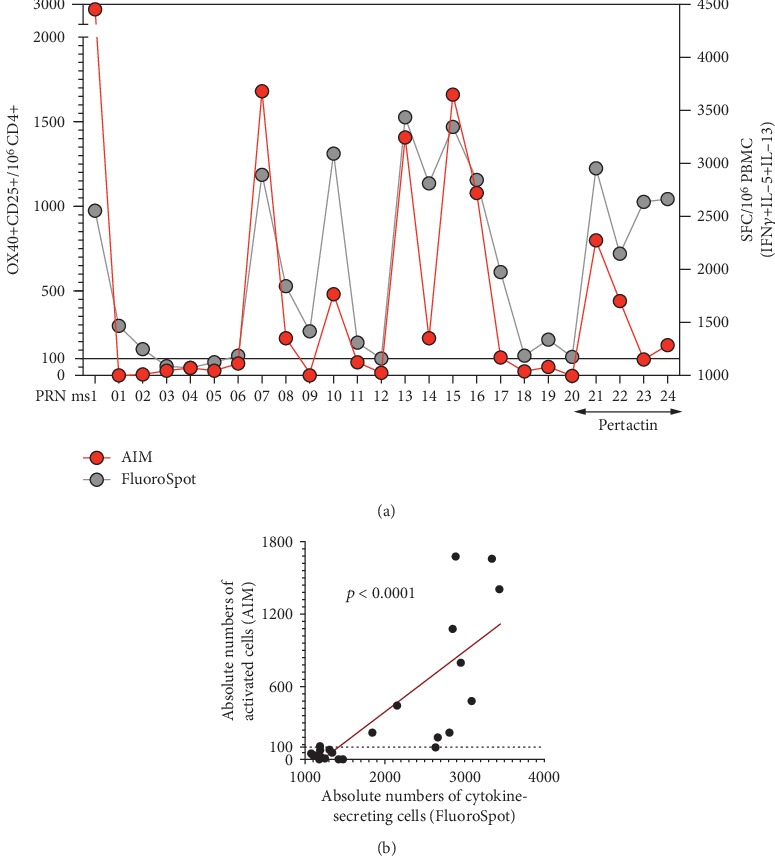
Correlation of results of the ex vivo AIM assay and 14 days restimulation assays. (a) The data show the overlay of the average of the individual peptide response as captured by either AIM or FluoroSpot assays for a selected mesopool deconvolution from a representative donor. (b) The best fit of the peptide data set is represented by a linear regression line (red) and the *p* value expresses Spearman's rank correlation coefficient test.

The results presented above indicate that both the in vitro restimulation*/*FluoroSpot and the ex vivo AIM assays are suitable methodologies for the large-scale BP screen. Given that both assays are suitable, we next addressed the question of whether the two different assay platforms would also lead to the identification of similar epitopes. When compared, it was found (Figure 7) that the two results obtained in the in vitro restimulation/FluoroSpot and the ex vivo *AIM* assays were highly correlated (*p* < 0.0001). In general, the correlation between the two assays is high. However, it is also noted that in some instances (Figure 7(a)), peptides 10, 14, 23, and 24 are associated with high activity in the FluoroSpot assay and relatively low AIM signal. It is not clear whether this is simply within experimental error or corresponds to a true reproducible phenomenon. In any case, it is noted that in the vast majority of instances, the peptides associated with high FluoroSpot activity are still positive for the specified AIM assay threshold. Since our concern is to identify an assay to be used as primary screen, those peptides would still be identified as positive and characterized in more detail in secondary assays, regardless whether the FluoroSpot or AIM would be used as a primary screen. Overall, based on these results, it appears that the two platforms are equivalent and potentially interchangeable.

## 4. Conclusions

The results of this study demonstrate that both the in vitro restimulation/FluoroSpot and the ex vivo AIM assays are suitable methodologies for the large-scale BP screen. In general, both assay platforms yield reproducible results when the assays are repeated in independent experiments performed on different days, and these findings have been verified utilizing different independent donors. Based on the results, we tentatively set a threshold of 100 cells and SI of 2 to identify positive MS candidates for deconvolution.

Surprisingly, our results indicate that aP donors also exhibited equivalent memory T cell reactivity against the ACT antigen, which is not found in the aP vaccines. Higher levels of antibody against ACT have also been observed in the plasma of aP-primed donors (unpublished observations). Thus, aP-vaccinated subjects are associated with immune responses against a variety of BP antigens not just present in the aP vaccine, which is consistent with the notion that aP prevents disease but not BP colonization/exposure [18, 19, 21].

Because both assay platforms appear to yield equivalent results, the question arises related to which of the two might be preferable. The ex vivo AIM platform is more laborious on the assay day (but this step can be at least partially automated), while the in vitro restimulation step, by definition, has a longer time to completion and requires more steps. The ex vivo AIM assay has three important advantages, as (1) it is not dependent on the responding cell to secrete any particular cytokine, (2) it introduces minimal physiological perturbations, as compared to a 2-week culture period, and (3) it allows mapping of subcellular populations and further phenotypic characterization [42].

Finally, in terms of biological relevance, both assay platforms correctly reidentify known antigens such as pertactin as an important source of BP epitopes and in addition identify novel epitopes derived from novel antigens such as D-alanyl-D-alanine transpeptidase and siroheme synthase. These results further support the interest and feasibility of performing a whole-genome-wide screen of BP T cell antigens. Both the in vitro restimulation/FluoroSpot and the ex vivo AIM assays results are associated with the expected polarization patterns, further underlining the biological relevance of the observations. In an age of increasingly reported cases and outbreaks of whooping cough throughout the globe, along with imperfect protection provided by currently administrated aP vaccines, the identification of novel antigens associated with BP virulence and persistence is warranted and crucial for better control and prevention of both transmission and disease.

## Data Availability

All The data used to support the findings of this study are included within the article or included within the supplementary information associated with this manuscript.

## Supplementary Materials

Supplementary Materials Supplementary Table 1: characteristics of the donor population. Supplementary Table 2: list of individual peptides of each megapool. Supplementary Table 3: list of antibodies used in the study.
